# systemPipeR: a multipurpose workflow management system for reproducible data analysis

**DOI:** 10.1093/nargab/lqag032

**Published:** 2026-04-02

**Authors:** Le Zhang, Daniela Cassol, Brendan Gongol, Thomas Girke

**Affiliations:** Institute for Integrative Genome Biology, University of California, Riverside, 1207F Genomics Building, Riverside, CA 92521, United States; Institute for Integrative Genome Biology, University of California, Riverside, 1207F Genomics Building, Riverside, CA 92521, United States; Institute for Integrative Genome Biology, University of California, Riverside, 1207F Genomics Building, Riverside, CA 92521, United States; Institute for Integrative Genome Biology, University of California, Riverside, 1207F Genomics Building, Riverside, CA 92521, United States

## Abstract

Workflow management systems (WMS) are essential for creating and automating multi-step data analyses and ensuring the reproducibility of biological insights. Although numerous WMS solutions exist, few provide deep integration of command-line software with the R and Bioconductor ecosystems, where a substantial portion of statistical modeling and downstream scientific analysis is performed by a large user base. systemPipeR addresses this gap by offering a unified environment that links R-based analytical steps with command-line tools through a standardized workflow specification. It enables the design and execution of reproducible workflows on both local and high-performance computing systems, while allowing users to select the most appropriate R or command-line tool for each analysis step. The latest version introduces a fully redesigned architecture that streamlines workflow construction, execution, monitoring, and reporting. Key enhancements include a flexible workflow management class object, integration of the Common Workflow Language (CWL), formal declaration and standardized execution of both R and command-line steps, utilities for metadata management, and automated generation of scientific and technical reports. Together, these advances establish systemPipeR as a general-purpose R-based WMS for building and executing end-to-end workflows for reproducible analysis of complex data in genomics and other data-intensive fields. The software is distributed as a free open-source Bioconductor package (https://bioconductor.org/packages/systemPipeR).

## Graphical Abstract

**Graphical Abstract:**
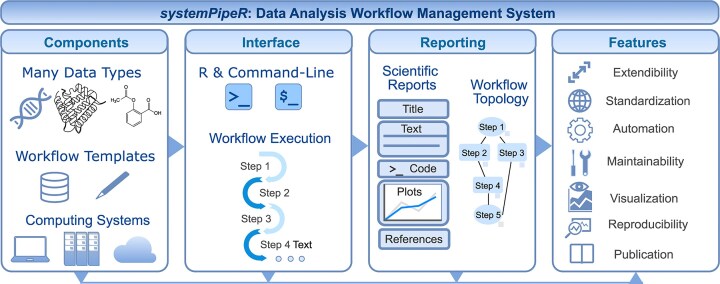

## Introduction

Many biological and biomedical fields are becoming increasingly data-driven, posing major challenges in the analysis of both small- and large-scale datasets. This shift is driven by the rapid growth of scientific data and the widespread adoption of high-throughput technologies in genomics, proteomics, and metabolomics, producing experiments with ever-increasing resolution, from tissues and organs to single cells [1, 2]. Not only are datasets growing in size and number, but their complexity and heterogeneity are also rapidly increasing. The analysis of such data typically requires multiple processing steps, each involving dedicated algorithms or software tools. To ensure reproducibility in these multi-step analyses, standardized workflows are essential. These workflows chain analysis steps in a compatible order, beginning with raw data and passing intermediate results sequentially through downstream stages.

Workflow management systems (WMSs) are essential for designing, customizing, and executing such workflows efficiently [3, 4]. They provide flexibility in how diverse data types can be analyzed reproducibly, which is increasingly important given the heterogeneity of modern datasets and the pace at which new analytical methods emerge [5, 6]. A well-designed WMS should address most of the following key requirements. First, *abstraction* and *standardization* ensure that data processing steps are described consistently under controlled syntax rules. Second, *automation* enables workflows to run reliably from start to finish, minimizing human error and promoting time efficiency. Third, *modularity* and *robustness* support checkpointing and enhance failure tolerance. Fourth, *scalability* allows workflows to accommodate increasing data volumes and more sophisticated analysis strategies. Fifth, *portability* across personal computers, high-performance computing (HPC), and/or cloud systems makes it possible to process large or computationally demanding datasets that exceed desktop capabilities. Sixth, *versioning, reusability*, and *shareability* facilitate collaboration and broader community adoption of well-defined workflows. In practice, achieving full coverage of the above capabilities is an ongoing optimization process. No WMS is likely to satisfy every requirement to its fullest extent, particularly because workflows rely on diverse software tools that themselves vary in maturity, performance, and reproducibility. Nevertheless, using systems that meet the majority of these requirements substantially enhances *reproducible* data analysis, which is essential for transforming biological data into reliable knowledge that supports informed decision-making across genomic, environmental, agricultural, and biomedical sciences. Moreover, the degree requirements such as cutting-edge scalability or robustness are needed depends heavily on specific research requirements: some applications demand exceptionally large-scale, distributed execution, whereas others benefit more from flexibility and tight integration with familiar analytical environments such as R.

Several WMSs are available across diverse data analysis domains, each offering distinct design principles. Graphical user interface–based systems include Taverna [7], Pegasus [8], Galaxy [9], and KNIME [10]. Domain-specific language (DSL) systems include Nextflow [11, 12] and Snakemake [13]. A third category consists of standardized specifications like the Workflow Description Language (WDL) [14] and the Common Workflow Language (CWL) [15, 16], which separate definition from execution to enable portability. Although all these systems enhance reproducibility [17], their practical value is subject to the specific needs of the user, particularly regarding whether they emphasize extreme scalability, robustness, or the flexibility offered by interactive and graphical working environments.

Despite this diverse landscape, a notable gap remains within the R and Bioconductor ecosystems. R is a premier environment for statistical modeling and visualization [18, 19], yet few WMSs successfully unite these capabilities with external command-line software. Existing R-native solutions typically specialize in one of two directions: they either focus on pure R reproducibility without dedicated interfaces for external tools [20, 21], or they focus on wrapping command-line tools without a framework for declaring native R analysis steps [22]. Consequently, researchers often face a trade-off between the interactivity of R and the robust management of external software.

To address this, *systemPipeR* provides a unified environment that integrates command-line tools and R-based analytics within a single framework. Its architecture coordinates raw data processing, statistical modeling, and automated reporting without requiring users to leave the R ecosystem. A detailed comparison of how *systemPipeR* complements existing WMSs is provided in the “Results and Discussion” section.

**Figure 1.**
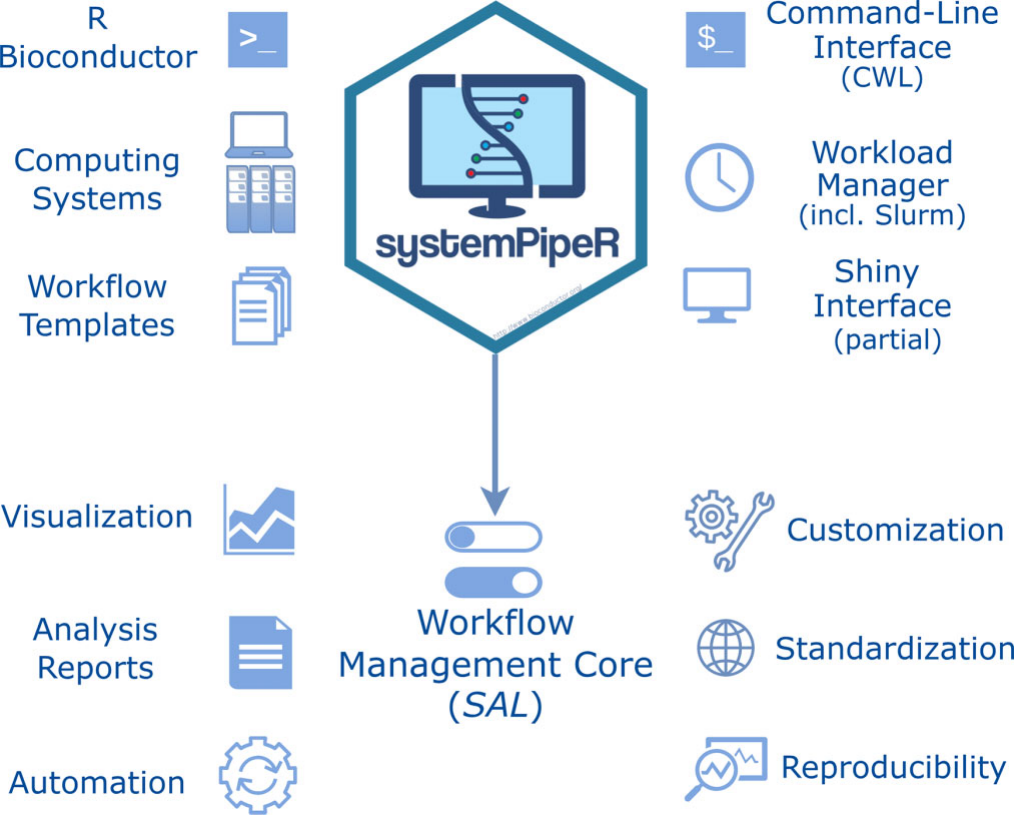
Overview of *systemPipeR’s* functionalities. *systemPipeR* is a multipurpose data analysis WMS that unifies R with command-line tools. The CWL community standard is integrated to describe and run command-line software. Central to the environment is a workflow management class object, termed *SAL* (short version of *SYSargsList*). It defines all information relevant for running workflow steps, status tracking, visualizing workflow topology graphs, and creating downstream analysis reports. The environment can be run on personal computers and HPC systems. *systemPipeR* takes advantage of widely adopted data structures and functionalities within the Bioconductor ecosystem. Numerous predefined workflow templates provide a user-friendly starting point for a wide range of data analysis needs. A graphical interface, developed as a separate Shiny app (*systemPipeShiny*), is also available.

**Figure 2.**
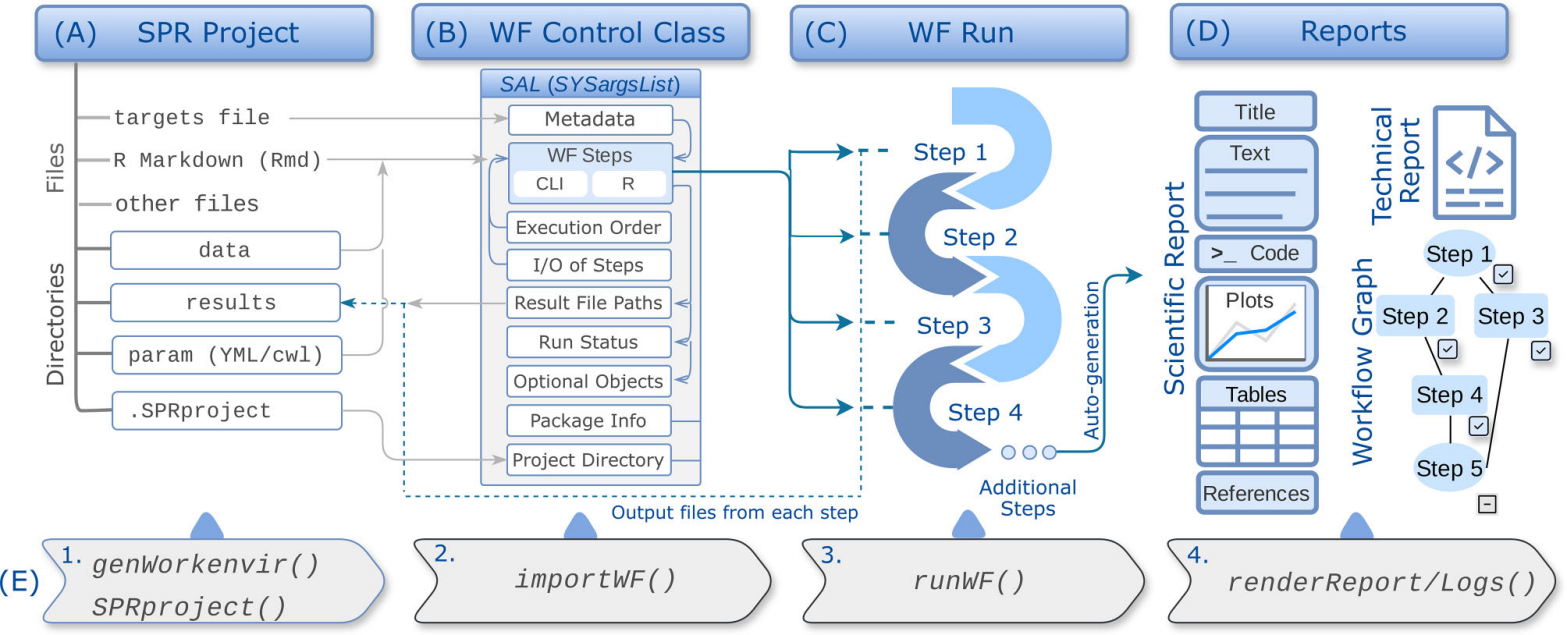
Design overview of the *systemPipeR* (SPR) WMS. The workflow organization is divided into four stages: (**A**) *Project Initialization*: The root directory organizes all necessary input data, metadata (targets), and parameters. The *Rmd* file serves as both the workflow definition and the source for the final scientific report. This environment is auto-generated by the functions listed in (E1). (**B**) *Workflow Control*: Instructions and metadata are loaded from the project environment into the *SYSargsList* (*SAL*) S4 class. This object acts as the central engine, managing dependencies, command-line arguments, and execution order. Additional details on *SAL* are provided in Supplementary Fig. S1. Connections among *SAL* slots and how they are populated from files under (A) are indicated by arrows; only the most important connections are shown for clarity and brevity. (**C**) *Execution*: The workflow is executed via *runWF*. During this phase, input/output connections are resolved automatically, and run status is auto-saved to the *.SPRproject* directory to allow for tracking and restarts. (**D**) *Reporting*: Upon completion, the system generates technical and scientific reports (derived from the *Rmd* source), as well as interactive topology graphs illustrating run statistics. (**E**) The primary command sequence (1–4) required to initialize, load, and run a workflow, as well as generate its reports.

This article presents the new version of the *systemPipeR* WMS (Figs 1 and 2). The initial version of this package was developed for constructing and running data analysis workflows with an emphasis on next-generation sequencing (NGS) applications [23]. While it provided useful functionality for integrating R with command-line software, its design limited the creation of new and increasingly complex workflows. These limitations have been resolved in the fully revised version of *systemPipeR* (V2), which incorporates a complete redesign and numerous new features. Key enhancements include: (i) adoption of the CWL community standard for describing and executing command-line software; (ii) introduction of a flexible and easy-to-use workflow management and control object; (iii) efficient management of metadata; (iv) an expanded reporting infrastructure supporting both technical and publication-quality scientific reports; (v) implementation of an interactive workflow topology viewer; and (vi) many additional functionalities supporting reproducible research. The following sections describe the design and unique features of the new version of *systemPipeR*, and a step-wise workflow run-through example showcases its functionalities for automated and reproducible data analysis.

## Materials and methods

### Implementation overview

*systemPipeR* is implemented as an open-source software package using the R programming language for statistical computing and graphics and is freely available from Bioconductor and GitHub [24, 25]. The package follows the design principles of the R and Bioconductor community for developing reusable, extensible, and maintainable data analysis software. The general design principles of the package are detailed in the “Materials and methods” section. Its functionalities, usage, and applications are discussed in the “Results and Discussion” section, while further technical information about classes, associated functions, and configuration options is provided in the Supplementary data of this article, as well as the frequently updated tutorials on Bioconductor.

### Objects and classes

*systemPipeR* adopts Bioconductor’s S4 class system for defining novel objects while leveraging existing classes widely used in genomics and related fields. These objects and associated methods provide a standardized framework for defining and managing workflows. Individual workflow applications incorporate other R and Bioconductor packages, as well as external command-line software. Common classes used in *systemPipeR* workflows include *SummarizedExperiment, AnnotationDb, DNAStringSet, GRanges*, and *TxDb* [25, 26].

Central to *systemPipeR*’s WMS is the workflow management class object *SYSargsList* (*SAL*). *SAL* is a list-like S4 object designed to organize all components needed to define, execute, and track workflows and to generate downstream data analysis reports. Its structure is illustrated in Figs 1 and 2 and Supplementary Fig. S1. It stores information about workflow steps (R and command-line steps), their dependencies, sample and experiment metadata, input/output files, and reporting instructions. *SAL* thereby serves as a central control structure that links workflow step definitions, data, and documentation. A complete list of core classes, methods, and functions defined by *systemPipeR* is given in Supplementary Tables S1–S3.

### R and command-line steps

Workflow steps in *systemPipeR* can consist of either command-line or R steps. For command-line steps, *systemPipeR* uses CWL [15] as the backend to describe commands in a standardized and declarative manner. CWL instructions are processed and integrated into *systemPipeR’s SYSargs2* objects, which define command-line steps within *SAL* (Fig. 2 and Supplementary Figs S1 and S2). This organization captures the command-line syntax, parameter settings, and paths to input/output files associated with each step. Additional details about the integration of CWL are provided in the Supplementary Methods Section S1.

To process R code chunks as workflow steps in a similar way, *systemPipeR* introduces the *LineWise* class, which encapsulates R code as workflow steps managed by *SAL* (Fig. 2 and Supplementary Fig. S1, and Section S2.3). This design allows users to convert R code into workflow steps without first wrapping the code as standalone command-line tools, thus substantially lowering the barrier for including R-based analytics in workflows for non-expert users. Where preferred, R-based command-line tools can still be incorporated and used in the same framework. The combined use of *SYSargs2* and *LineWise* provides a uniform workflow design and execution model in which R and command-line steps coexist in a central workflow class.

### Importing and running workflows

Fully populated SAL instances, including all analysis steps of a workflow, can be generated by importing them with a single command from R Markdown (or R) source files that incorporate both narrative text and code sections (Fig. 2 and Section S2.3). Code chunks annotated with *systemPipeR*-specific options define the command-line and R steps in a workflow and are automatically registered as workflow steps during import. A set of dedicated functions and methods (Supplementary Tables S1–S3) provides a high-level interface for designing, controlling, executing, and monitoring workflows. For example, applying a single execution command to a *SAL* object can run an entire workflow or a selected subset of steps, and a subsequent command can generate the corresponding workflow reports, including scientific and technical reports.

To support different computing loads, *systemPipeR* provides several parallelization options that can exploit multiple CPU cores and nodes on personal computers or HPC systems. The logical definition of the workflow remains unchanged across these environments, while resource usage and parallelization parameters can be tuned for individual steps or globally to the available infrastructure. Detailed execution modes and parallelization options are described below and in Sections S2.5 and S3.

### Workflow reports

After a workflow is executed, a comprehensive data analysis report can be generated from the *SAL* workflow object or directly from the corresponding R Markdown source file. This flexible, dual approach allows for rendering a scientific report based on the original Rmd version used when the workflow was loaded, or producing interactively modified versions that incorporate user updates. This human-readable and visually informative report automatically embeds relevant scientific results, such as tables, plots, and images. The visualization tools within systemPipeR utilize JavaScript-based engines like *htmlwidgets* and *Graphviz* to embed interactive workflow topology graphs and run statistics directly into reports. In addition to the scientific report, a technical report is also generated. This report provides critical run statistics, including system logs and the processing status of individual input samples for each workflow step. More technical details concerning report generation and customization are available below and in Section S2.8.

### Design features

*systemPipeR*’s object-oriented design provides a consistent user interface across workflows, simplifying adoption and everyday use. By organizing workflow definitions, metadata, and reporting instructions within a single class object, the framework supports transparent, end-to-end documentation of analyses. The use of S4 classes and modular design facilitates long-term maintainability and extensibility, allowing new functionalities to be added without breaking existing workflows. This is particularly important in fast-evolving domains such as genomics, where new tools and analysis strategies are frequently introduced.

## Results and discussion

### Overview

The new version of *systemPipeR* (V2) has been implemented as a multipurpose WMS and reporting environment that unifies R with command-line tools [23]. It enables scientists to analyze a wide range of data types—from small exploratory datasets to large-scale omics experiments—on personal computers or HPC systems with a high level of reproducibility, customizability, and extensibility (Figs 1 and 2). At its core, *systemPipeR* integrates CWL for describing command-line steps [15] and R-based steps with the *SAL* workflow management object, which coordinates R- and command-line-based steps under a shared management and tracking model. This architecture allows users to choose, for each analysis step, the optimal R or command-line tool while keeping workflow logic, metadata, and reporting tightly synchronized.

The generic design of *systemPipeR* enables users to run existing workflows, customize them, or design entirely new ones while taking advantage of widely adopted data structures within the Bioconductor ecosystem. Workflows can be imported from R Markdown documents that combine narrative descriptions with R and CWL-based code chunks, making it straightforward to define complete workflows and, at the same time, generate fully documented analysis reports. In addition, the environment supports both end-to-end and partial execution of workflows, with built-in restart capabilities that avoid redundant computation and facilitate long-running analyses.

A key goal of the redesign was to move beyond pipeline orchestration and provide an integrated environment that spans from raw data processing to high-level statistical modeling and interpretation. To this end, *systemPipeR* introduces a set of functionalities that are rarely found together in a single WMS: (i) hybrid R/CLI workflow definitions based on CWL and R Markdown; (ii) a workflow management class object (*SAL*) that stores workflow steps, dependencies, metadata, and reporting instructions; (iii) automated scientific and technical report generation; (iv) interactive visualization of workflow topology and run status; (v) a graphical user interface to a subset of *systemPipeR’s* functionalities; and (vi) a collection of ready-to-use workflow templates for common omics applications. Below, we highlight these components in more detail and discuss how they distinguish *systemPipeR* from other WMSs.

### Integration of R with command-line tools

Many bioinformatics workflows depend on a combination of command-line tools and R-based analyses. For example, command-line tools are often used for read mapping, quantification, sequence assembly, or variant calling, whereas R is typically used for statistical modeling, visualization, and downstream interpretation. A central design objective of *systemPipeR* was to support this hybrid model directly from R without requiring users to switch to other environments.

By using CWL as a backend [15], *systemPipeR* can describe command-line tools and workflows in a portable, declarative format that is compatible with community standards (Section S1). These CWL descriptions are imported into R as *SYSargs2* objects and managed as workflow steps within *SAL*. In parallel, R-based analysis steps are encapsulated in *LineWise* objects, which allow R code to be treated as workflow steps without requiring conversion to command-line wrappers (Fig. 2B and Section S2.3). As a result, users can define workflows that interleave command-line and R steps, while dependencies, input/output connections, and metadata are handled uniformly by *SAL*.

This hybrid execution model is particularly valuable for research workflows that need to combine established command-line tools with rapidly evolving R-based methods. It enables transparent tracking of both types of steps, facilitates reuse of CWL definitions from external sources, and supports flexible reruns and modifications as analytic strategies evolve. Additional implementation details on CWL integration, command-line rendering utilities, and automatic CWL generation from command-line strings are provided in the Supplementary data (Supplementary Figs S2–S4).

Python-based tools can also be incorporated as command-line workflow steps alongside native R-based analyses via *systemPipeR’s* command-line execution framework and CWL-based workflow definitions. When tighter coupling is required, Python code can also be integrated directly within R workflow steps using established interoperability tools such as *reticulate* [27].

### Metadata

Efficient management of metadata is a central requirement for correct interpretation of data and analytical results, as incomplete or inconsistent annotations can lead to misinterpretation and compromise reproducibility. Metadata typically include dataset identifiers, sample names, experimental conditions, data provenance, and a variety of other descriptive attributes. In *systemPipeR*, metadata are stored in dedicated slots of *SAL* objects and are most conveniently imported from a tabular *targets* file at workflow initialization (Fig. 2A and B and Supplementary Fig. S2). Support for additional community standards, such as the *SummarizedExperiment* class, is also provided [25]. Sample labels defined in the metadata are automatically propagated to downstream analyses, where they are used to annotate plots, tables, and output files (Fig. 2D). This systematic inheritance of labels ensures consistent linkage between samples, results, and associated files across workflow steps, reducing the likelihood of manual errors and maintaining coherent naming conventions throughout the analysis and reporting process.

### Workflow definition and management

*systemPipeR* leverages R Markdown as a high-level interface for defining and documenting workflows (Fig. 2 and Supplementary Fig. S4). Alternatively, workflows can also be specified directly in R scripts if preferred. R Markdown documents typically contain narrative text interleaved with code chunks. In *systemPipeR*, selected code chunks are annotated to be interpreted as workflow steps and imported into *SAL* with a single command, either all at once or interactively, one step at a time. Command-line-based steps are defined by referencing CWL parameter files, while R steps are wrapped in *LineWise* constructors (Supplementary Fig. S4).

Once imported, the *SAL* object provides a centralized management hub of the entire workflow, including step dependencies, metadata of input data and experimental designs, and output locations. Users can execute the full workflow, run only selected steps, or restrict runs to a subset of input samples in respective steps. The state of a workflow run, including completion status and runtime information for each step and sample, is automatically recorded in the project directory (*.SPRproject* folder) and within *SAL*. This enables workflows to be paused and resumed, rerun selectively, or transferred to other systems while preserving their status.

The tight integration between R Markdown and *SAL* has several advantages. First, it keeps code, narrative descriptions, and results in one place, improving transparency and user control. Second, it allows the same R Markdown document to serve as both workflow specification and scientific report template, reducing duplication and potential inconsistencies. Third, it provides a natural path for extending workflows with additional steps or refactoring them as new methods become available.

### Visualizing workflow topologies and run status

**Figure 3.**
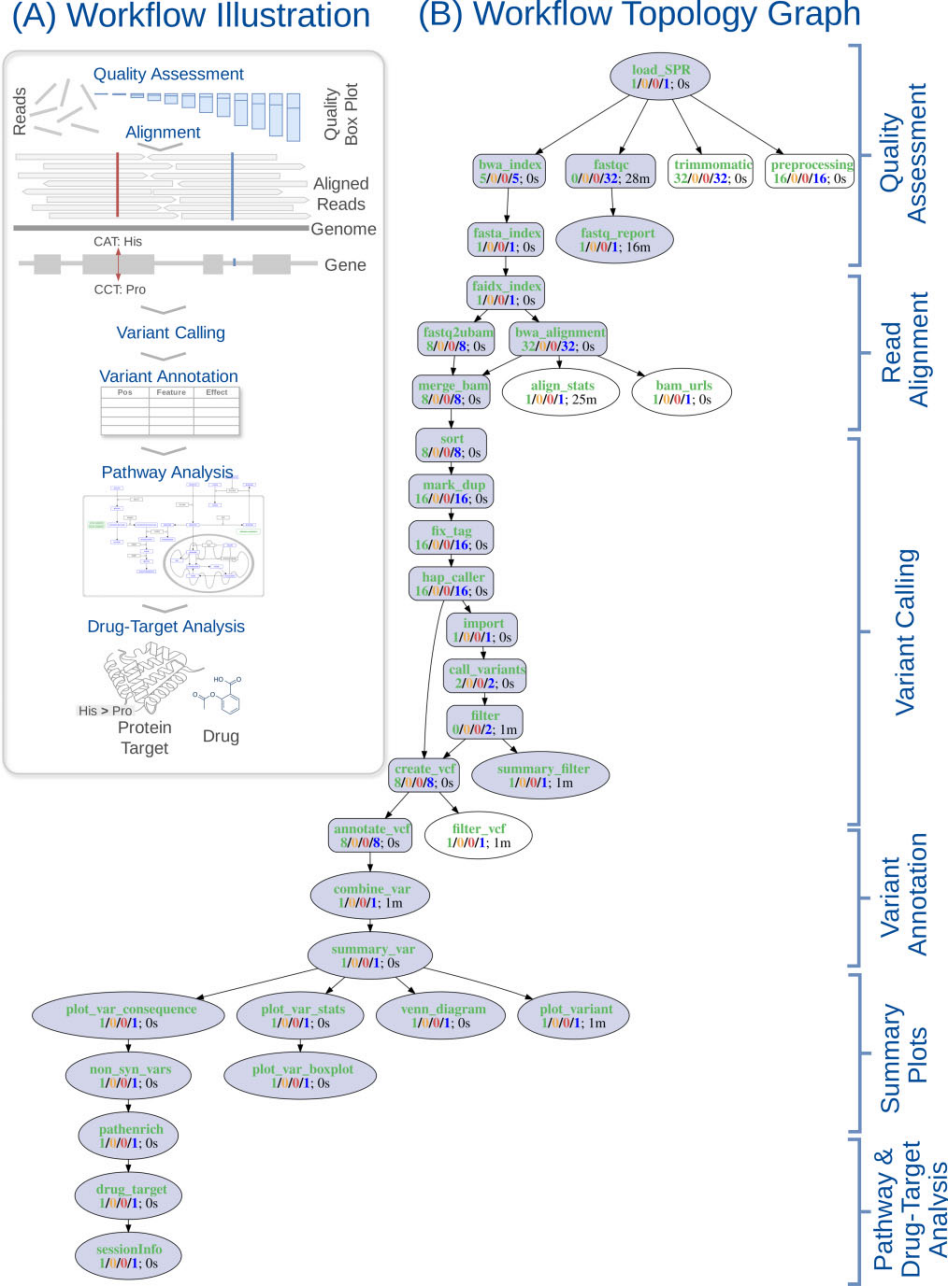
Illustration and topology graph of a multi-modal VAR-Seq workflow comprising 35 steps. (**A**) A high-level visual summary illustrating the main analytical components of the workflow, including read quality assessment, alignment, variant calling, functional annotation, pathway analysis, and identification of drugs targeting proteins affected by predicted functionally relevant genetic variants. (**B**) The corresponding workflow topology graph generated with *systemPipeR*’s *plotWF* function. The graph is organized as a directed acyclic graph (DAG), where nodes represent discrete analytical steps and edges denote execution dependencies. Function arguments allow rendering the topology in alternative layouts. Vertex colors and shapes encode execution status at the level of workflow steps and input samples. The graph is interactive; for example, hovering over a node reveals detailed sample-level and timing information, and zoom functionality is supported.

Understanding and debugging complex workflows requires insight into both their structure, and success of individual processing steps and runtime. To support this, *systemPipeR* provides visualization tools that generate interactive workflow topology graphs directly from *SAL* (Figs 2 and 3). The *plotWF* function renders nodes (workflow steps) and edges (dependencies) and annotates each node with color-coded status indicators summarizing the processing state of each step’s inputs (e.g. pending, running, successful, warning, or error). Users can zoom into specific regions, inspect tooltips with sample-level information and runtime statistics, and export plots in multiple graphics formats. These topology graphs can be embedded into both scientific and technical reports, as well as into web applications such as Shiny. Beyond visualization, *systemPipeR* maintains detailed status tracking information accessible through a set of accessor functions (Supplementary Table S2). This facilitates monitoring progress in large workflows, identifying failure points, and programmatically summarizing results across steps and samples.

### Scientific and technical reports

*systemPipeR* creates scientific and technical reports, each describing different aspects of workflow runs (Fig. 2D). The *Scientific Reports* describe how a data analysis was performed, the generated results, along with detailed information for interpreting the results in a comprehensive and visually informative document. These reports resemble a research paper, where user-generated text is combined with analysis results. This includes support for citations, auto-generated bibliographies, code chunks with syntax highlighting, and inline evaluation of variables to update text content. Data components in a report, such as tables and figures, are automatically embedded and updated when a document is rebuilt or a workflow is rerun. If needed, the workflow topology maps, described in the previous section, can be included in scientific analysis reports. Since *systemPipeR’s* built-in reporting mechanism is automated, it increases the reproducibility of the generated results and saves scientists a substantial amount of time and effort generating high-quality analysis reports, thus shortening the time and effort from data analysis to preparing manuscripts for publication. The associated *Technical Report* provides relevant logging information generated during the corresponding workflow run on a system. It allows to inspect executed command-line commands, software versions, messages, warnings, and errors. Both report types can be dynamically generated in HTML, PDF, or other formats during or after workflow execution and can be updated without re-running all computations. Together, they ensure that methodological details and analytical outputs remain tightly linked, reinforcing best practices in reproducible research [28].

### Parallelization and deployment

To handle increasing data volumes and computational demands, *systemPipeR* supports a range of parallelization and deployment options (Section S3). Workflows can be executed in serial or parallel mode on a single machine or distributed across multiple nodes on HPC clusters and cloud platforms. Parallelization is implemented mainly via the *batchtools* and *BiocParallel* packages [29, 30], which support a variety of HPC schedulers and queueing systems (e.g. Slurm). Resource requirements such as CPU cores, memory, and wall time can be specified for individual workflow steps and stored in *SAL*, allowing the same logical workflow to be adapted to different computing environments. Users without access to HPC infrastructure can still execute the same workflows in serial or modestly parallel modes on personal computers, while maintaining compatibility with deployments on large-scale computing systems.

### Workflow templates for typical applications

For efficient use of *systemPipeR* without designing custom workflows from scratch, the affiliated *systemPipeRdata* package (also available from Bioconductor) provides a collection of workflow templates for typical applications. With a single initialization command, users can generate fully populated workflow project instances containing the workflow script (R Markdown), example input data, and parameter files required to execute a selected workflow (Fig. 2A). This allows users to rapidly test workflows, adapt existing templates for new analyses, or develop new workflows based on established structures.

Many templates are ready-to-use end-to-end workflows, requiring only the substitution of user-provided input data to run complete analyses. They are accompanied by comprehensive documentation describing common experimental designs, analysis strategies, and parameter choices at each step. In addition to application-specific workflows spanning domains such as omics analyses (RNA-Seq, ChIP-Seq, VAR-Seq, Ribo-Seq, etc.), general sequence analysis, and cheminformatics, the package includes a workflow skeleton that provides a general outline for developing custom analyses tailored to specific use cases.

All templates are intentionally modular and extensible. Users have the flexibility to use them as they are, or adjust parameters, and modify or extend individual workflow steps to meet their analytical requirements (Fig. 2A). While these templates provide practical and well-documented starting points, *systemPipeR* does not aim to curate or maintain a comprehensive workflow repository for community submissions. Establishing and sustaining such a resource would require ongoing validation, quality control, and methodological review beyond the scope of this project. Instead, *systemPipeR* emphasizes enabling users to build workflows either from scratch or by revising existing templates, while preserving methodological ownership at the level of individual users and research groups.

### Graphical user interface

The companion package *systemPipeShiny* extends the framework with a Graphical User Interface (GUI), offering intuitive web-based access to selected *systemPipeR* functionalities. Unlike static reports, the Shiny environment remains reactive: users can adjust parameters to instantly update tables and visualizations without re-executing upstream workflow steps. Additionally, *systemPipeShiny* includes a graphics workbench for assembling publication-ready figures through interactive post-processing. The application can be deployed locally or on centralized servers for remote access. Additional details are provided in the package vignette on Bioconductor.

### Use case example

The following use case illustrates core functionalities of *systemPipeR* using a *multi-modal VAR-Seq workflow* as an example (Fig. 3A). This workflow—provided by *systemPipeRdata*—integrates cohort-level variant analysis with hypothesis-generating drug discovery routines. It was selected because it represents a realistic, non-linear analysis scenario with broad applicability. The workflow combines scatter/gather execution, heterogeneous command-line and R-based steps, and downstream multi-modal analyses, thereby demonstrating how *systemPipeR* manages workflow complexity and execution dependencies.

The major components of the workflow and their dependencies are summarized in the topology graph shown in Fig. 3B, which provides a compact representation of execution order, branching structure, and step-level dependencies within the workflow.

#### Workflow setup, execution, and monitoring

The workflow is initialized through a setup function that utilizes a pre-configured template. This function automatically generates a standardized project directory structure, which is populated with parameter files and the necessary input data download information for demonstration purposes (Fig. 2A). These test datasets can be replaced with user-provided data for real analyses. Experimental metadata are specified in a tabular *targets* file that records sample identifiers, input file paths, and additional metadata required for the analysis. Workflow outputs are written to a centralized *results* directory, and an executable R Markdown (Rmd) file is provided as a template for defining the analysis and generating reproducible scientific reports.

Workflow steps defined in the Rmd file are imported into a *SAL* workflow management object that captures all analysis steps and their dependencies (Fig. 2B and C). A workflow execution function (*runWF*) enables execution of the complete workflow or selected subsets of steps. Workflow structure and execution status can be inspected via the *SAL* object or visualized as a directed acyclic graph (Fig. 3B). The same workflow specification can be executed on local systems or HPC environments by providing appropriate resource parameters.

#### Overview of analysis steps

Users may execute the workflow as provided or customize individual R- and command-line-based components (Fig. 3). For example, specific tools such as aligners or variant callers can be substituted with compatible alternatives by updating paths to the corresponding CWL parameter files in the Rmd file.

Raw sequencing reads undergo quality assessment to evaluate sequencing performance and identify technical artifacts such as low base quality, adapter contamination, or compositional biases [31]. Optional read preprocessing steps, including adapter and quality trimming [32, 33], are supported but not required by default, allowing users to follow recommended best practices for downstream variant calling while retaining flexibility.

Reads are aligned to a reference genome using a variant-tolerant aligner (e.g. BWA-MEM) [34, 35], producing alignments that accommodate mismatches and small indels to optimize read placement. Variant discovery is performed using the Genome Analysis Toolkit (GATK) [36–38] following a gVCF-based strategy. GATK HaplotypeCaller records genotype likelihoods for both variant and non-variant genomic regions, enabling accurate discrimination between homozygous reference sites and missing data. Individual gVCF files are aggregated and jointly genotyped at the cohort level to produce a unified multi-sample VCF with improved calling consistency. Jointly called variants are subsequently filtered using cohort-level quality metrics to retain high-confidence calls suitable for downstream interpretation.

High-confidence variants are functionally annotated with respect to genes, transcripts, and predicted coding consequences, representing a key multi-modal integration step that maps genetic variation to biological entities [39, 40]. Annotated variants are aggregated across samples to support cohort-level comparisons, estimation of variant frequencies, and identification of shared or sample-specific patterns. Summary statistics and visualizations provide a concise overview of variant composition and functional impact across the cohort.

To illustrate how variant analysis outputs can inform early-stage drug discovery, the workflow includes a hypothesis-generating extension that integrates genetic, biological, and pharmacological modalities. Variants are aggregated at the gene level to quantify functional variant burden across the cohort, followed by pathway enrichment analysis to identify affected biological processes. Prioritized genes and pathways are then mapped to known drug targets and associated compounds, enabling the identification of drugs that target proteins with predicted functionally relevant genetic variation.

#### Workflow reporting

All analysis steps are documented in an automatically generated workflow report (Fig. 2D) that summarizes results, figures, and execution metadata. Reports include tables and plots generated during workflow execution, links to large result files, and code blocks capturing the exact commands used for each step. Software versions and parameter settings are recorded to support reproducibility, and report sections are updated automatically when workflows are re-executed or modified.

Overall, this use case demonstrates how *systemPipeR* supports end-to-end automation of complex data analysis workflows, from initial setup and execution to final reporting. The workflow design emphasizes modularity, reproducibility, and flexibility, allowing users to substitute tools, adjust parameters, and extend analyses across multiple analytical modalities. By supporting local and HPC within a flexible analytical environment, *systemPipeR* accommodates diverse data scales with varying computational requirements.

### Choosing between different WMSs

A diverse ecosystem of WMSs has emerged across bioinformatics, reflecting varied architectural priorities. The following comparison contrasts *systemPipeR* with two widely used non-R and two R-based systems to highlight its complementary features (Table 1). This overview identifies where *systemPipeR* offers unique functionality rather than duplicating existing capabilities. It is not intended as a ranking or exhaustive feature evaluation, as such in-depth comparisons are beyond this article’s scope and would quickly become outdated given the rapid evolution of these tools. Furthermore, while many additional excellent WMSs are currently available, a complete survey is not provided here; readers are referred to recent reviews and community perspectives for broader benchmarking of the field [41].

**Table 1.** R-centric comparison of key capabilities distinguishing *systemPipeR* from other established workflow frameworks

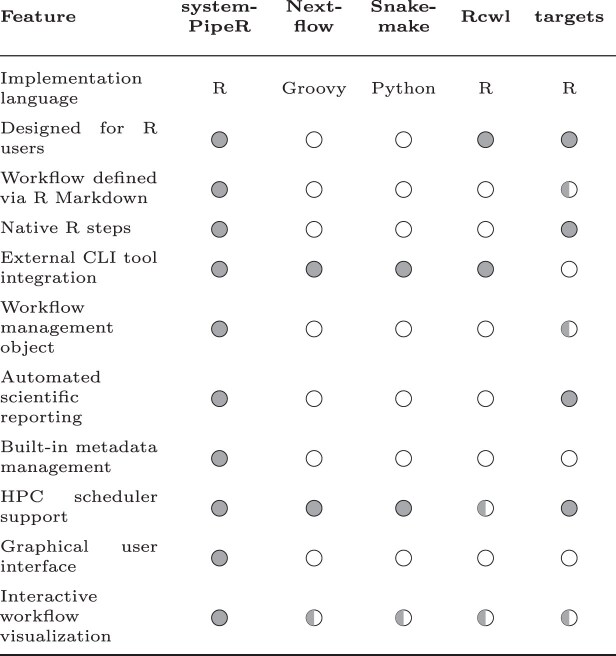

This comparison highlights how *systemPipeR* bridges the gap between scalable command-line execution and native R-based statistical analysis. Note that the other listed WMSs possess additional unique strengths not detailed here, as an exhaustive comparison is beyond the scope of this overview. Meaning of symbols: 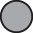 = Strong; 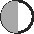 = Partial; 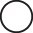 = None.

*Nextflow* and *Snakemake* are established standards for production-grade pipelines requiring high portability across local, HPC, and cloud infrastructures [12]. *Nextflow* is a dataflow-oriented WMS that uses a Groovy-based DSL to decouple workflow logic from the execution environment, facilitating massive parallelization. Conversely, *Snakemake* is a file-oriented WMS that uses a Python-based DSL to infer execution through pattern-matching and explicit dependency rules. While both excel at managing high-throughput compute tasks, they are generally less optimized for workflows requiring seamless, bidirectional integration with an interactive R environment. In particular, these systems are well suited for extreme-scale, production-oriented workflows that prioritize maximal throughput, robust fault tolerance under highly distributed execution, and long-term pipeline deployment independent of interactive data analysis.

Among R-based systems, *targets* (successor of drake) provides a declarative framework for pure R workflows, offering dependency tracking and seamless R Markdown integration [20, 21], though it lacks a dedicated interface for external command-line tools. Conversely, *Rcwl* connects R with CWL to standardize command-line execution [22, 42], but focuses less on declaring native R steps.

Within this landscape, *systemPipeR* bridges batch-oriented computation and interactive data analysis by integrating command-line and R-based workflow steps within a single framework. External tools can be executed on local systems or HPC resources, while statistical modeling, visualization, and automated reporting are performed seamlessly within R. While *Nextflow* and *Snakemake* are preferable for extreme-scale, production-oriented pipelines, and *targets* is well suited for pure R dependency management, *systemPipeR* provides a cohesive solution for workflows in which command-line-based data processing must connect directly to complex statistical interpretation and reporting in R. A distinctive feature is its ability to define and document workflows directly within R Markdown, unifying workflow specification, analysis, and reporting in a single, reproducible format. The framework is also designed to be accessible to non-expert users who need to develop custom workflows.

A distinct advantage of WMSs operating within an interactive environment such as R is the flexibility to iteratively interact with and refine data analyses. Users can respond immediately to intermediate results by adjusting parameters, generating diagnostic plots, or adding analysis steps during the same session. This immediacy supports rapid hypothesis testing and real-time exploration—capabilities that are often less accessible in non-interactive systems. While careful management is required to preserve robustness when workflows evolve in an ad hoc manner, this flexibility is essential for many discovery-oriented research settings. Consistent with this utility, Bioconductor download statistics indicate broad adoption: *systemPipeR* is among the most frequently downloaded Bioconductor packages, with 50 681 recorded total downloads in 2025 and 13 695 downloads from unique IP addresses.

## Conclusion

The *systemPipeR* package unites R and Bioconductor software resources with external command-line tools to standardize and automate a wide range of data analysis tasks. Its functionality reduces the complexity and time required to translate both small- and large-scale data into interpretable research results, while a built-in reporting framework improves reproducibility by automatically generating publication-quality analysis reports. The software implements a novel object-oriented workflow control object that provides a flexible interface for designing, modifying, and executing workflows. At its backend, the widely adopted CWL standard is used to describe command-line steps in a uniform format supported across multiple programming languages. Workflow topology graphs and technical run reports enable efficient monitoring of execution status and workflow outcomes. In addition, an intuitive graphical user interface offers interactive access to selected *systemPipeR* functionalities and supports visual exploration and interpretation of results. The framework provides sufficient flexibility to select optimal software for individual workflow steps, customize existing workflows, and design new ones. Pre-configured workflow templates are included for several data analysis applications. Together, these features establish *systemPipeR* as a versatile workflow management system for designing and executing complex, reproducible data analyses.

## Supplementary Material

lqag032_Supplemental_File

## Data Availability

*systemPipeR* and *systemPipeRdata* are freely available for all common operating systems from Bioconductor and GitHub here: https://bioconductor.org/packages/systemPipeR and https://bioconductor.org/packages/systemPipeRdata/. The corresponding DOI instances on Figshare are https://doi.org/10.6084/m9.figshare.29936933 and https://doi.org/10.6084/m9.figshare.29936936.
