# Supplementary material for: systemPipeR: a multipurpose workflow management system for reproducible data analysis
Source: NAR Genom Bioinform. 2026 Apr 2;8(2):lqag032. doi: 10.1093/nargab/lqag032 (PMC13044936; doi:10.1093/nargab/lqag032)
Supplement: lqag032_Supplemental_File [file lqag032_supplemental_file.pdf]

Supplement for *"systemPipeR: A Multipurpose Workflow Management System for Reproducible Data Analysis"*

Supplementary Methods

The following Supplementary Methods section describes the technical details of *systemPipeR's* implementation and usage, complementing the conceptual overview in the main manuscript. It expands on the structure and behavior of the underlying workflow management class components, command-line integration mechanisms, execution strategies, reporting infrastructure, and extensibility features. While some features are reintroduced for context and clarity, the content below focuses largely on implementation details not covered in the main article. Additional technical documentation is available in the *systemPipeR* vignettes on Bioconductor and GitHub.

S1 Integration of CWL

The new version of *systemPipeR* adopts CWL to describe command-line tools and workflows in a declarative, portable, and reproducible manner. CWL uses YAML syntax [1] to specify command-line calls. YAML is a widely used human-readable standard that is often employed to write configuration files. Most commonly, CWL specifies command-line calls with two files (Figures S2A-B): a step or workflow definition (cwl) file and a configuration (yml) file. The step definition file contains the necessary information to construct a command-line call including its parameters. The values assigned to the parameters can be provided by the configuration file (Figure S2B). For efficient parameter management, *systemPipeR* extends this functionality by allowing the transfer of parameter values from an easy-to-maintain tabular file, termed *targets* file, to the configuration file (Figure S2D). The use of the targets file is optional, but greatly simplifies the management of parameters and input data. When used, the parameters defined in the *targets* file will be processed automatically, eliminating the need for users to manipulate the yml files directly or write complex loops. This way, all input files (*e.g.* biosamples) listed in a *targets* file can be processed automatically either in sequential or parallel mode (see below for parallelization options). Internally, the parameter values in the *targets* file are assigned to the parameters with matching names in the yml file (Figure S2C & D). This parameter passing is initialized by variable names.

S1.1 Rendering Command-Line Calls from CWL Files

systemPipeR offers several key features for building and troubleshooting workflows. One such feature is a command-line rendering function, which produces command-line strings or calls for workflow steps using *targets* metadata and CWL parameter files before execution (Figure S2C). A command-line string, in this context, is an instance of running software with specific parameter settings based on its command-line syntax. The ability to generate corresponding command-line strings for each input data set and parameter setting is invaluable for inspecting and testing workflow steps, for instance, by running any command-line calls defined by the workflow's CWL files from a terminal or web-based interface.

S1.2 Auto-Generation of CWL Files from Command-Line Strings

Auto-generation of CWL parameter files is another useful utility provided by *systemPipeR* (Figure S3). Here, users can simply provide the command-line syntax for running a new software to automatically generate the corresponding CWL definition and configuration files (cwl and yml). Alternatively, this operation can be performed stepwise in an interactive manner, where one can first generate, for a base command-line syntax, the corresponding CWL definitions. Next, one can modify and extend this CWL instance as needed until the desired result is achieved. Finally, the resulting CWL syntax can be written to CWL files or directly incorporated into *systemPipeR's* workflow class object (see *SAL* below). The auto-generation of CWL parameter files minimizes the burden of manually creating CWL files. This functionality is implemented in *systemPipeR's* *createParam* function group. For convenience, *systemPipeR* projects contain a collection of ready-to-use CWL parameter files for widely used bioinformatics tools. Users can directly use them to run preconfigured workflows or design custom workflows. Most CWL parameter files from external

community collections are also compatible and can be used in *systemPipeR* without any or only minor modifications.

S2 Interface for Managing and Running Workflows

For designing and executing workflows, *systemPipeR* provides a WMS that integrates R with CWL. This includes options to seamlessly combine R with command-line steps, while also taking advantage of R/Bioconductor's existing classes for large-scale data analysis, as well as a rich ecosystem for statistical data modeling, visualization, and report generation.

S2.1 Overview of Running Workflows

Running workflows in *systemPipeR* is straightforward (Figure 2). First, a workflow project environment is created using an initialization command (Figure 2E1). Next, a chosen workflow can be run from start to finish using only two commands, including a workflow load and a run command (Figure 2E2-3). After partially or entirely completing a workflow run, a detailed scientific analysis report can be generated by a third command (Figure 2E4).

S2.2 Workflow Management Class

systemPipeR introduces an object-oriented environment for managing, executing, and monitoring data analysis workflows. The core of the environment is the *SAL* workflow management object (an S4 class) along with a series of methods operating on *SAL* instances, such as constructors, accessors, and rendering functions (Figures 2B and S1). *SAL* provides a flexible workflow control center with a standardized interface to most functionalities in *systemPipeR*, including import and export, execution, status control, and visualization of workflows, as well as generation of scientific and technical reports.

The *SAL* class stores all the necessary information required to execute data analysis workflows (Figure 2). This includes the code for running each analysis step, how the workflow steps are connected, their inter-dependencies, and metadata describing the input data. Workflows can contain data analysis steps involving command-line software or R code. Each of the two types is managed by its own subclass that is a component of *SAL*. The classes *SYSargs2* and *LineWise* are for command-line- and R-based steps, respectively. Dependency tree validation methods are employed to evaluate the integrity of workflows. The step dependency tree defines the relationships between analysis steps, and thus the order they need to be executed. For visualizing workflow topologies and their dependency trees, the *plotWF* function can be used (see below for details).

S2.3 Initializing Workflows from R Markdown

A particularly intuitive and powerful option for running existing or designing new workflows in *systemPipeR* is via R Markdown (Figure 2A & D). This system allows to load of all analysis steps of a workflow with a single command into *systemPipeR's SAL* object for managing, executing, and monitoring workflows. R Markdown source files are composed of two main section types: narrative text and code chunks. During the rendering process of an R Markdown, scientific plots and tables are generated by the code chunks in real-time, and then inserted in the final PDF or HTML reports. In *systemPipeR*, workflow steps can be defined by the code chunks of an R Markdown. The code chunks can consist of either pure R code or command-line steps defined by the corresponding CWL instructions.

This design makes it very easy for users to run workflows and create reproducible data analysis reports at the same time. The entire analysis and reporting process requires users to execute only three functions (Figure 2B-D). First, an import function will load the entire workflow defined by an R Markdown into a *SAL* object. Next, the instructions in *SAL* are used to run the workflow, and finally the analysis report is generated based on information in *SAL* and the results generated by the workflow. The option of combining analysis code with the results that the code generates, makes R Markdown a powerful tool for reproducible research and data analysis. Additional advantages of defining *systemPipeR* workflows in R Markdown include increased transparency and reproducibility by keeping the code and results of an analysis connected, while the narrative text can provide valuable context information necessary for understanding the individual analysis steps, and for interpreting the generated analysis results. Moreover, R Markdown reports can be rendered into many static or dynamic file formats that are widely used for scientific publications and presentations.

The following provides additional details on how workflow steps are defined in *systemPipeR* using R Markdown. For declaring workflow steps, *systemPipeR* introduces *'spr'* as a single new argument under the option section of the code chunks in R Markdowns (Figure S4). The setting *'spr=TRUE'* declares a new workflow step. During the loading process of a workflow from an R Markdown file, only code chunks with this flag are registered as workflow steps. Other code chunks

lacking this flag can still be evaluated during report generation using the standard R Markdown code chunk options. When declaring command-line- and R-based workflow steps, two separate functions are used within code chunks that are termed *SYSargsList* and *LineWise*, respectively. The inputs required for the *SYSargsList* function are the paths to the CWL files that define a command-line step, and optionally the path to a *targets* file defining additional variables. The *LineWise* function enables R code blocks to be registered as workflow steps without requiring conversion of R code into external command-line tools. To declare an R workflow step, the corresponding code is encapsulated within a *LineWise* function call. R workflow steps are executed within a dedicated workflow R environment that is separate from the user's interactive R session. This separation prevents unintended naming conflicts or object overwriting during interactive development, while allowing workflows to be paused and resumed with a consistent execution state. When a workflow is restarted, the workflow environment—including objects and functions created by previous workflow steps—is restored within the workflow context. Within this workflow environment, R steps intentionally share state. This design enables reuse of intermediate objects and supports seamless integration of statistical modeling, visualization, and reporting across workflow steps, which is central to *systemPipeR*'s support for interactive, analysis-centric workflows in R. Users retain explicit control over environment boundaries and may selectively transfer objects between the workflow and interactive contexts. *systemPipeR* does not create a separate R environment or R process for each individual R workflow step. While this shared-environment model may be less suitable for workflows requiring strict isolation (e.g., when incompatible package versions are needed), it reflects a deliberate design choice aligned with common R analysis practices. For use cases requiring stronger isolation, the following approaches are recommended: (i) intermediate objects can be written to and reloaded from result files to avoid reliance on shared in-memory state; (ii) package conflicts can be mitigated by importing specific functions rather than attaching entire packages using the *package::function* syntax; and (iii) if necessary, R code can be encapsulated and executed as R-based command-line tools.

S2.4 Constructing and Modifying *SAL* Instances

SAL workflow instances can be constructed by loading workflow steps one at a time, or all steps in batch mode by importing them from a workflow script, usually an R Markdown. The stepwise approach is useful for customizing workflow steps, or developing and testing new workflows. For performing step-level modifications, *systemPipeR* provides a series of functions (Tables S1, S2 and S3) that allow users to adjust individual steps on the fly. When modifying workflows, the dependencies and connectivities between workflow steps can sometimes break, especially when working with complex *SAL* objects. If this happens, validity checking utilities will alert users, pinpoint the corresponding problems in SAL objects, and suggest solutions to correct them. After a workflow has been tested and optimized, it is recommended to perform the actual production data analysis by running a workflow from start to finish in fully automated mode. This is critical to minimize human error and maintain a high level of reproducibility.

S2.5 Running Workflows

Running workflows in *systemPipeR* is not only straightforward, but also flexible. It supports both end-to-end and partial execution of workflows, where users can choose to process all input datasets (samples) or only a subset of them. Restart functionalities are provided that make it easy to re-run all or individual analysis steps, or to continue runs without repeating already completed steps to save time. The following describes important options for running workflows in more detail.

Executing Entire Workflows. Running entire workflows in *systemPipeR* from start to finish is simple by applying its *runWF* function to a fully populated *SAL* workflow object. Usually, this is done without specifying parameters that restrict the run to a subset of workflow steps or input samples.

Executing Selected Steps. For partial runs, users can select workflow steps under the *'steps'* argument of the *runWF* function. When making step selections, it is important to avoid dependency violations caused by the specific selection of steps, such that the input of a chosen step does not exist, or an upstream step generating this input is missing or has not been executed yet. The option of executing specific workflow steps is useful for many use cases, including the development of new analysis steps and performance optimization.

Execution of a Subset of Input Datasets. Users can choose to run workflows only on a subset of input datasets. This is useful when dealing with large numbers of input datasets where initial tests on a subset of them can help estimate computational resource requirements or identify formatting problems in the input data.

Forced Re-execution of Steps. The completion status of workflow steps and their associated input data is recorded during workflow runs (see the following section for details). To maximize processing performance, already completed results are not unnecessarily re-computed by default, unless a user chooses to overwrite this behavior by enforcing the re-execution of specific steps and datasets, regardless of their completion status.

Restarting Workflow Runs. If a workflow run is interrupted or needs to be paused, the exact state of the corresponding *SAL* object is automatically saved and can be used to resume a workflow run from the point where it stopped. This restart functionality is extremely useful for completing workflows involving long run times without unnecessarily repeating already completed steps. Importantly, the same behavior allows users to rerun a workflow only on newly obtained input datasets, without repeating the processing with already completed samples.

Reinitializing Workflows. To restart workflows from the very beginning without including any previous run history, users can simply reinitialize their workflow project and *SAL* object. This is sometimes required after configuring and testing a workflow, and subsequently running it from the same system location to generate the final production results in fully automated mode.

Porting Workflow Projects to Other Systems. If needed, partially or fully completed workflows can be moved with or without their input and result data to other computing systems (*e.g.* personal computers, cluster or cloud systems). This is useful for archiving, continuing, or re-executing workflows on other systems. During a workflow run, operations already performed are stored in a specific project directory, termed *'.SPRproject'* (Figure 2A). The run information stored in this directory is required for documentation purposes as well as restarting workflows on other systems. Moreover, users can export a specific *SAL* instance, created during a workflow run, to a new R Markdown file (Table S3). This way, a specific run instance can be preserved and reloaded on another system. This is useful for sharing specific workflow runs with collaborators or the community.

S2.6 Status Tracking of Workflow Runs

The ability to track and report the status of workflow runs is an essential feature of WMSs. This involves detailed monitoring of the progress of every input dataset in each workflow step, along with execution messages, such as warnings and errors. *systemPipeR* collects this tracking information in real time in its project directory and *SAL* object instances. Two main options are provided for viewing and accessing the status tracking information of a workflow. First, a summary of this tracking information can be viewed in an easy to read color-coded layout, where each status type (pending, running, and completed) is represented by its own color. This feature is provided by the summary view (print) method of the *SAL* object. Simply typing the name of a *SAL* object will return its current summary view. Alternatively, more detailed information can be extracted programmatically using several accessor functions that return the tracking information for each workflow step stored in *SAL* (Table S2).

S2.7 Visualizing Workflow Topologies and their Run Status

systemPipeR workflows can be visualized with the *plotWF* function (Figures 2D and 3). This versatile workflow viewing function accepts a *SAL* instance as input and returns the corresponding visual workflow topology map that offers many interactive features. Each plot contains the following information: (i) topology graph of a workflow depicting the dependencies among steps; (ii) run status of workflow steps, including pending, running, success, errors and warnings; (iii) processing status statistics of input data; and (iv) run time for processing each input data set. Workflow topology maps are useful during the design and testing process of workflows, as well as after running a workflow to inspect the success of each step. In a workflow topology map, workflow steps and their dependencies are represented by nodes and edges, respectively. The nodes are labeled by the corresponding step names; and the two different node shapes, oval and rectangular, represent R and command-line steps, respectively. Run status information for each step is given by color-coded numbers printed inside the nodes. Workflow dependency maps can be embedded in workflow reports or saved to graphics files, where users can choose among a variety of formats (e.g. *png*, *jpg*, *svg* or *DOT*; Figure 3). Several interactive features are included in the workflow topology maps. These include interactive export to graphics files, zooming options, and mouse over to access detailed information about the samples and their run status (Figure 3). Most of these interactive features are maintained when embedding workflow topology maps in *systemPipeR's* technical and scientific reports saved to HTML files, or web applications such as *Shiny* (Figure 3).

S2.8 Report Generation

systemPipeR creates two types of reports, each describing different aspects of workflow runs (Figure 2D). The *Scientific Report* resembles a scientific publication describing how a data analysis was performed, the generated results, along with detailed information for interpreting the results in a comprehensive and visually informative document. The associated *Technical Report* provides relevant logging information generated during the corresponding workflow run on a system that is useful to assess the success of each workflow step and troubleshooting problems.

Scientific Reports. They can be auto-generated during or after workflow runs using R Markdown [2]. The structure of R Markdown documents and how they can be used to load workflows in *systemPipeR* is described above. The following focuses on how *systemPipeR* uses R Markdown as a reporting environment. In *systemPipeR*, R Markdown workflow documents are imported into its *SAL* workflow object. During a workflow run, the code sections of the imported R Markdown are evaluated and the corresponding data analysis results generated. At the same time, an analysis report is dynamically generated in HTML, PDF or other formats. If needed, a caching system can be enabled that allows updating reporting components without repeating unnecessary computations.

Technical Reports. Technical information generated during workflow runs is automatically collected in a central location within each instance of a *systemPipeR* project environment. After partial or full completion of a workflow, the logging information of a run is used to generate a technical report in HTML or other formats, which is also generated with R Markdown. It includes detailed system information, software execution commands, as well as messages, warnings, or errors issued during each workflow step. Easy visual navigation of *Technical Reports* is provided by including the corresponding topology graph of a workflow run (see above). The technical details included in these reports are important for assessing the success of each workflow step and to facilitate troubleshooting possible problems.

S3 Parallelization on Personal, HPC or Cloud Systems

When running data analysis workflows, users can substantially reduce their run times by taking advantage of parallel processing. For this, *systemPipeR* supports a wide range of parallelization concepts, including parallelization across multiple cores or CPUs and GPUs on single machines, several high-performance computing (HPC) nodes, and cloud systems. Often, parallelization allows completion of complex computations within hours rather than days or weeks, as can be the case for non-parallelized workflow runs. For users who do not have access to HPC systems, *systemPipeR* always provides the alternative to run the same workflows in serial mode using a single CPU core, or if possible in parallel mode across several CPU cores available on personal computer processors. The parallelization functionalities in *systemPipeR* are largely based on existing and well-maintained R packages, mainly *batchtools* and *BiocParallel* [3, 4]. By making use of cluster template files, most schedulers and queueing systems are also supported (*e.g.* Slurm, Torque, Altair, Sun Grid Engine). Users also have the flexibility to parallelize all workflow steps or only some of them.

To simplify the configuration and execution of workflow steps in serial or parallel mode, *systemPipeR* uses for both the same *runWF* function that is very easy to use. Parallelization simply requires appending of the parallelization parameters to the settings of the corresponding workflow steps, each requesting the computing resources specified by the user (*e.g.* amount of CPU cores, RAM, and run time). These resource settings are stored in the corresponding workflow step of the *SAL* workflow object. The processing of the affected step will be performed in parallel or serial mode depending on whether the parallelization settings are present or absent, respectively. If needed, users can enable and update the parallelization settings of workflow steps in *SAL* interactively (Table S3). For reproducibility and documentation purposes, however, it is usually preferred to explicitly include these parallelization instructions in the source R Markdown instance that is used for a specific workflow run.

S4 Workflow Templates

For efficient use of *systemPipeR* without designing custom workflows, the affiliated *systemPipeRdata* package (available from Bioconductor) provides a series of workflow templates. This data package allows generating workflow project instances with a single command that are fully populated with the corresponding workflow script, test data and parameter files required for running a chosen workflow (Figure 2A). With this, users can quickly test workflows, design new ones from existing templates, or create new ones from scratch. Many workflow templates are ready-to-use workflows (see *systemPipeRdata* package) where users only need to provide their custom input data to run an analysis from start to finish. This includes comprehensive documentation explaining the rationale for common experimental designs, analysis methods, and parameter choices for each step.

Figures

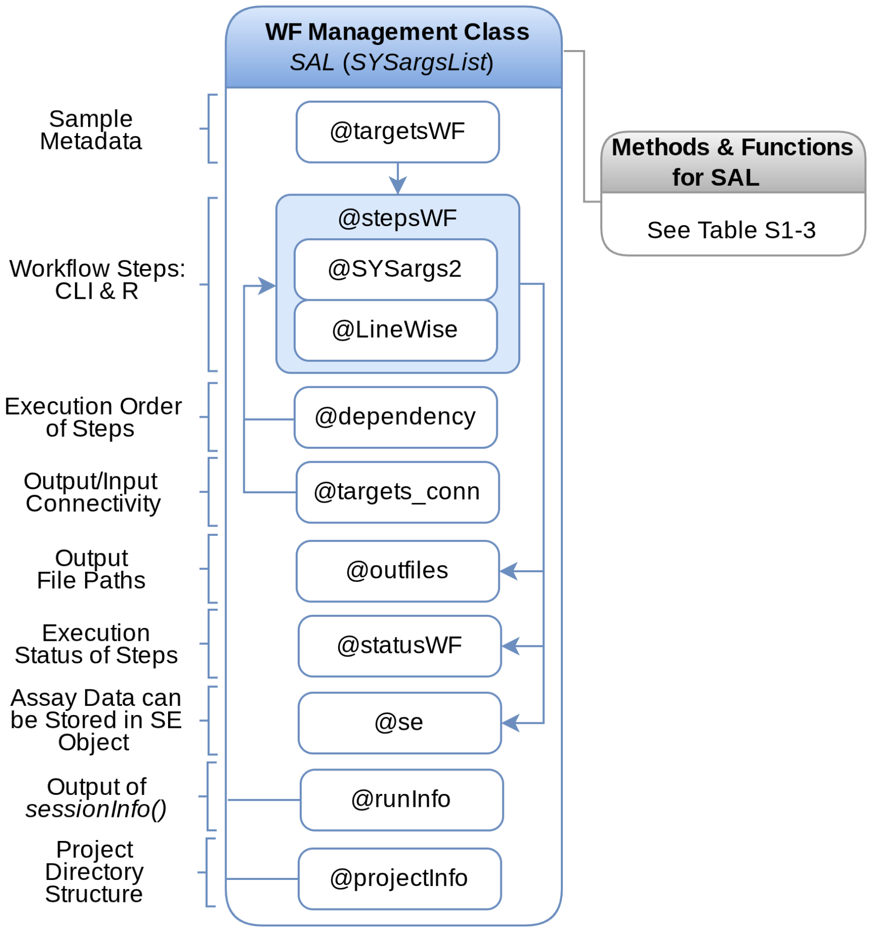

Figure S1: Structure of the *systemPipeR* Workflow Management Object. The *SYSargsList* (*SAL*) S4 class serves as the central hub for workflow management and execution. It organizes all relevant workflow components into distinct slots, described here in the order they appear in the illustration. 1. *targetsWF*: Stores the sample metadata provided by *targets* file in *DataFrame*. It acts as the initial input for the workflow. 2. *stepsWF*: The main object holding the list of all workflow steps. Each item in this list is either a *SYSargs2* object (for command-line tools) or a *LineWise* object (for R code). 3. *dependency*: Defines the execution order. It specifies which parent steps must be completed successfully before a specific step can begin. 4. *targets_connection*: Defines how data flows between steps. It tracks which output column from a previous step serves as the input column for the next step. 5. *outfiles*: A list containing the file paths of all result files generated by the workflow (*e.g.* BAM files). 6. *statusWF*: Tracks the state of every step (*e.g.* Pending, Success, Error) and includes execution time statistics. 7. *SE*: If needed *SummarizedExperiment* objects can be included here to hold numeric data (like gene count matrices) that are compatible with downstream Bioconductor analysis. 8. *runInfo*: Captures technical details for reproducibility, such as the R session information and the timestamp of the workflow initialization. 9. *projectInfo*: Stores the project's directory structure (paths to data, results, and param folders) and global configuration settings. Table S3 lists the methods for constructing, accessing, and viewing *SAL* objects, along with functions that operate on them.

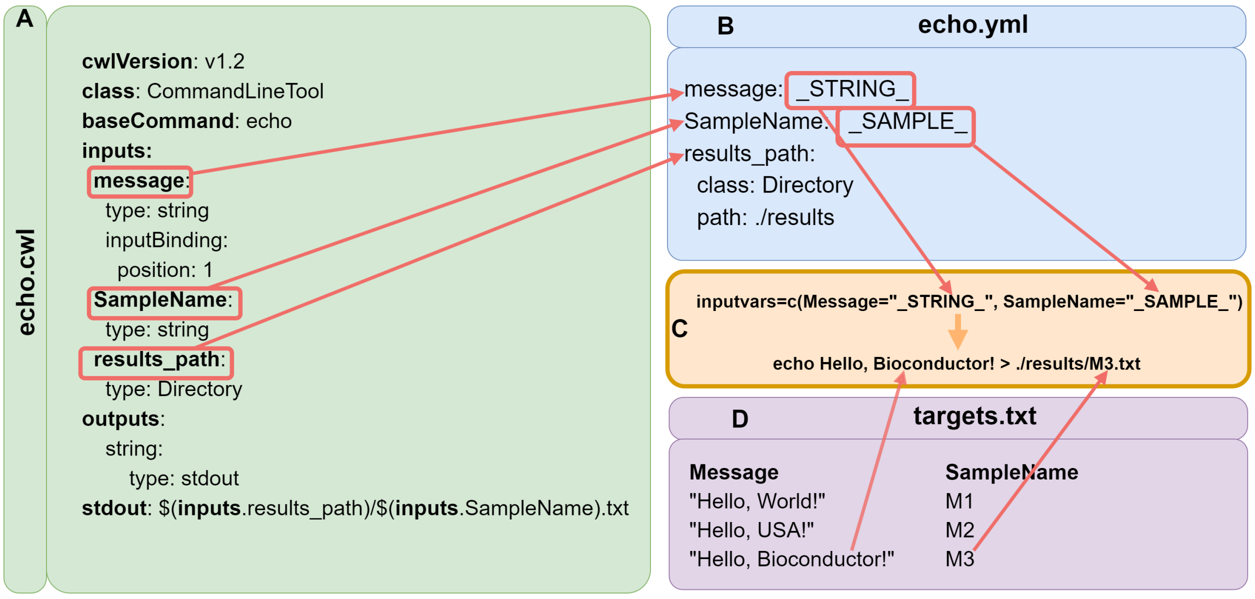

Figure S2: Example of describing a command-line step with CWL in *systemPipeR*. To define a command-line step, CWL uses a cwl step definition file (A) and a yml configuration file (B). The cwl file defines the command-line tool along with its parameters. The values assigned to the parameters can be stored in the yml file. The chosen example, uses the basic *'echo'* command on Linux systems that displays text assigned to an argument (variable). For convenience, *systemPipeR* allows passing of parameters from a tabular *targets* file (D) to the *yml* file. The *targets* file greatly simplifies the management of parameters and input data, but its usage is optional. When used, the parameter values in the *targets* file are assigned to the parameters with the same name in the yml file. The corresponding rendering function in *systemPipeR* constructs the proper command-line calls for each entry (row) in the *targets* file (C). In the chosen 'echo' example, the final command-line call is shown in panel (C) that corresponds to the last parameter value (3rd row) in the *targets* file.

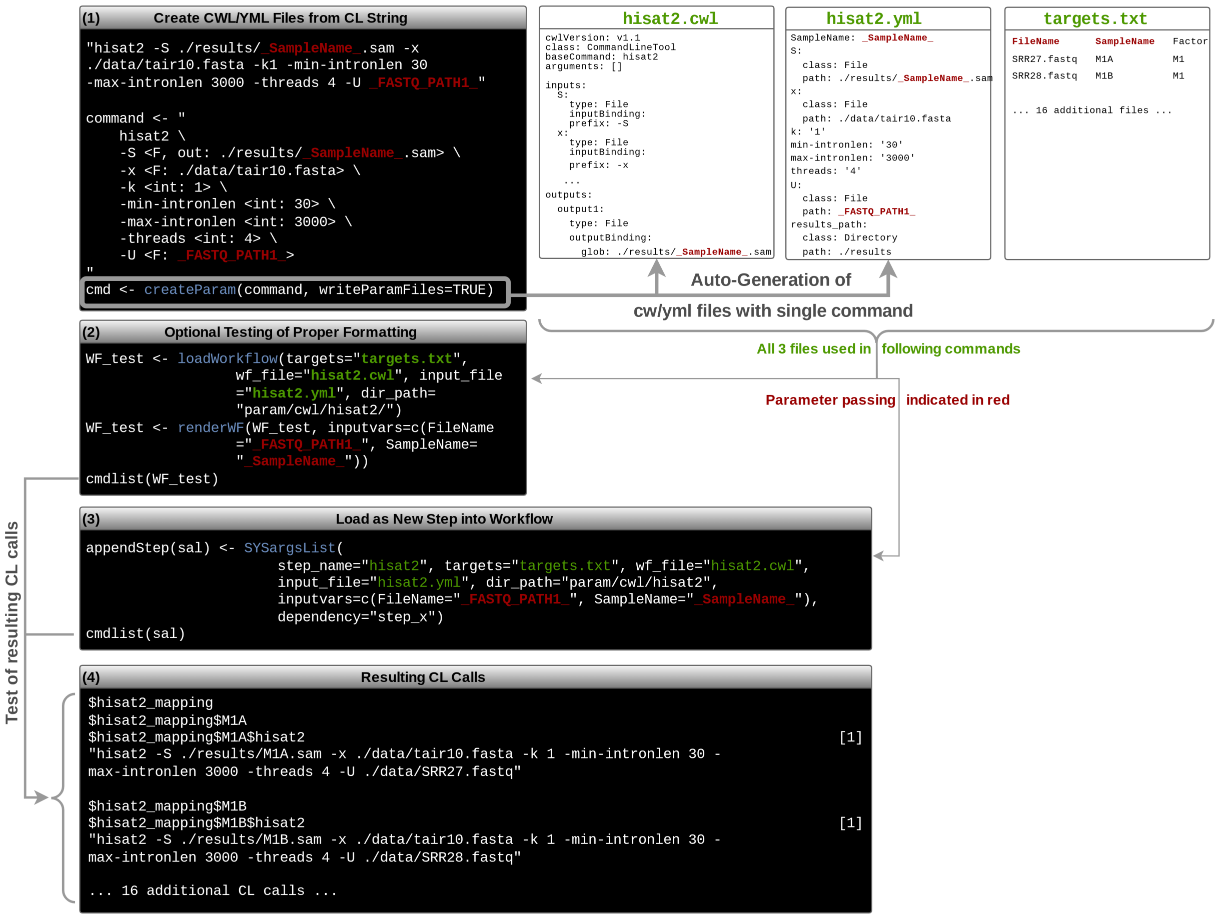

Figure S3: Auto-generation of CWL files from command-line (CL) string with a single command. (1) Example of a CL for running the HISAT2 RNA-Seq aligner. The top displays the original CL string for HISAT2, followed by the expected input format. The *createParam* function utilizes this CL to generate the corresponding parameter files: *hisat2.cwl* and *hisat2.yml* (shown on the right). (2) Combined with a *targets* file (here *targets.txt*), describing the experimental design, the functionality of the parameter files can be optionally tested by assembling the expected CL calls with the *cmdlist* function. (3) The new parameter files along with the *targets* file are used to define a step in a workflow, here alignment step. (4) Similarly as under (2), the *cmdlist* function can assemble CL calls using the *SAL* workflow control object as input. Green font is used to highlight how parameter files are incorporated into the corresponding function calls. The red color coding demonstrates the flow of parameters from the tabular *targets* file to the *yml/cwl* files and, subsequently, to the resulting CL calls.

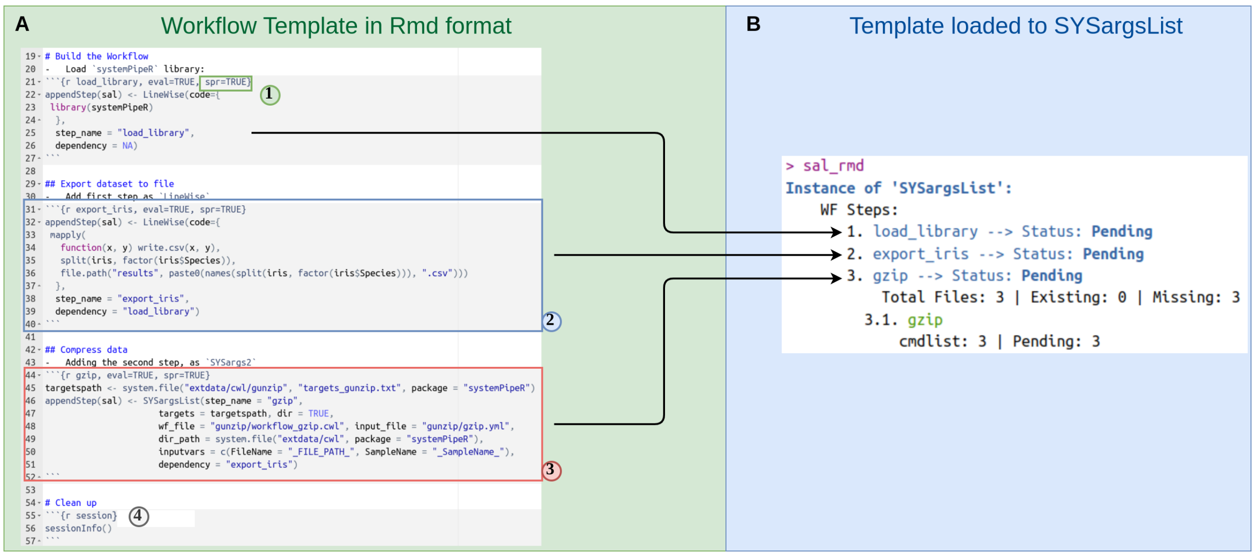

Figure S4: Illustration demonstrating how workflow steps are imported from R Markdown workflow files into *SAL* Workflow Management Object. (A) Workflow steps are declared in the header lines of the code sections (chunks) of R Markdown files. 1: Only code chunks with the setting *"spr=TRUE"* will be loaded into *SAL*. 2: Example of an analysis step with pure R code that will be loaded as a *LineWise* object into *SAL*. 3: Example of a CL step that will be loaded as a *SYSargs2* object into *SAL*. 4. Other code chunks lacking the *"spr=TRUE"* flag can be evaluated during report generation using the standard code chunk options of R Markdown. (B) The imported workflow steps are included in the summary view of the *SAL* object.

Tables

Table S1: Key methods for constructing and updating workflows within the *SAL* workflow control object. A comprehensive list of functions defined by the *systemPipeR* can be found in Table S3.

Methods	Level	Description
appendStep	Step	Adds a new step
replaceStep	Step	Replaces an existing step
yamlinput	Substep	Changes parameter settings of command-line tool(s)
dependency	Substep	Changes the step dependency of a step
updateColumn	Substep	Updates targets columns of a step
addResources	Substep	Adds computing resource requirements, such as number of CPU cores

Table S2: Important functions for extracting status information from *SAL* objects. For a more comprehensive list of accessor functions, consult Table S3.

Methods	Description
statusWF	List the processing status of workflow steps
targetsWF	Return the input and output *targets* instances
cmdlist	Return the command-line strings of workflow steps involving external command-line software
codeLine	Return the R code of *LineWise* steps
outfiles	List the paths to output files

Table S3: List of functions and methods defined by *systemPipeR* (not complete). As new functions continue to be developed, this list is expected to grow over time.

**Functions**	**Description**	**Class of input**
addResources	Add remote computing resources to a step	SYSargsList
alignStats	Alignment statistics	SYSargs/SYSargs2
appendCodeLine <-	Add a new R code line	SYSargsList/LineWise
appendParam	Accessories function to modify the Command-line	SYSargs2
appendStep <-	Add a new Step	SYSargsList
as.list	Coerce to list	Various objects
baseCommand	SYSargs2 accessor methods	SYSargs2
catlist	Accessor method	catDB
catmap	Accessor method	catDB
check.outfiles	Checking if the output files exist	SYSargs2/SYSargsList
check.output	Checking if the output files exist	SYSargs2/SYSargsList
clt	SYSargs2 accessor methods	SYSargs2
clusterRun	Submit command-line tools to cluster	SYSargs2
cmdlist	SYSargs2/SYSargsList accessor methods	SYSargs2/SYSargsList
cmdToCwl	SYSargs2 accessor methods	SYSargs2
codeChunkStart	LineWise accessor methods	LineWise
codeLine	LineWise/SYSargsList accessor methods	SYSargsList/LineWise
combineVarReports	Combine variant reports	Paths to VCF files
complexitylevels	Intersect levels of Venn diagrams	vennset
copyEnvir	Copy objects to new.envir	SYSargsList
cores	SYSargs accessor methods	SYSargs
countRangeset	Read counting for several range sets	BamFileList
createParamFiles	Construct SYSargs2 from command-line string	command-line string
dependency	SYSargsList/LineWise accessor methods	SYSargsList/LineWise
dependency<-	Replace dependency tree	SYSargsList
evalCode	Toggles option eval on the RMarkdown files	None
featureCoverage	Compute read coverage	BAM paths and Granges
featuretypeCounts	Read counts across feature types	BAM paths and Granges
files	Paths to CWL parameter files	SYSargs2 or LineWise
filterDEGs	Filter DEGs on stats	data.frame
filterVars	Filter VCF files	Paths to vcf files

Table S3: List of functions and methods defined by *systemPipeR* (not complete). As new functions continue to be developed, this list is expected to grow over time.

**Functions**	**Description**	**Class of input**
genFeatures	Generate ranges for variety of features	TxDb object
getColumn	Obtain paths to file from an earlier step	SYSargsList
getQsubargs	Arguments for qsub	Command of scheduler
goBarplot	Create bar plot of GOCluster_Report	data.frame
GOCluster_Report	Batch GO term enrichment analysis	list of gene IDs
GOHyperGAll	Performs over-representation analysis	character vector of IDs
GOHyperGAll_Simplify	Reduce redundancy in GO results	data.frame
GOHyperGAll_Subset	Subset GO result by GO IDs	data.frame
idconv	Feature ID conversion	data.frame
importWF	Import RMarkdown file as workflow	SYSargsList/LineWise
infile1	SYSargs/SYSargs2 accessor methods	SYSargs/SYSargs2
infile2	SYSargs/SYSargs2 accessor methods	SYSargs/SYSargs2
input	SYSargs2 accessor methods	SYSargs2
inputvars	SYSargs2 accessor methods	SYSargs2
intersectlist	Return pairwise intersects	INTERSECTset
intersectmatrix	Create intersect matrix	VENNset
linewise	LineWise accessor methods	LineWise
LineWise	Construct LineWise object	LineWise
loadWorkflow	SYSargs2 from CWL param and targets files	param file paths
makeCATdb	Generate gene-to-GO mappings and store as catDB	file path to GO annotation
mergeBamByFactor	Merge BAM files based on factor	SYSargs2
module	Interface to Environment Module system	action type
moduleAvail	List modules	none
moduleClear	Unload all modules	none
moduleInit	Load default modules	none
modulelist	List loaded modules	none
moduleload	Load specified software	name of software
modules	SYSargs/SYSargs2 accessor methods	SYSargs/SYSargs2
moduleUnload	Unload specified software	name of software
olBarplot	Bar plot for intersect sets	VENNset/INTERSECTset
olRanges	Range overlaps	IRanges and GRanges
other	SYSargs accessor methods	SYSargs
outfile1	SYSargs accessor methods	SYSargs
outfiles	SYSargsList accessor methods	SYSargsList
outpaths	SYSargs accessor methods	SYSargs
output	SYSargs2 accessor methods	SYSargs2
output_update	Updates the output files paths in the SYSargs2 object	SYSargs2
overLapper	Compute intersects among ID sets	list of ID vectors
plotfeatureCoverage	Plot results from featureCoverage	data.frame
plotfeaturetypeCounts	Plot results from featuretypeCounts	data.frame
plotWF	Plot workflow toplogy graph	SYSargsList
predORF	Predict ORFs	DNAString/DNAStringSet
preprocessReads	Run read pre-processing functions	paths to fastq files
printParam	Set of functions for modifying command-line	SYSargs2
projectInfo	Return project information	SYSargsList
qsubRun	Submit command-line tools to cluster (old)	SYSargs
readComp	Import sample comparisons from targets file	SYSargs/SYSargs2
reference	SYSargs accessor methods	SYSargs
renameParam	Accessories function to modify the Command-line	SYSargs2
renameStep <-	Rename workflow step	SYSargsList
renderReport	Render scientific report	SYSargsList
renderWF	Populate all the command-line in an SYSargs2 object	SYSargs2
replaceCodeLine¡-	Replacement R code	SYSargsList/LineWise
replaceParam	Accessories function to modify the Command-line	SYSargs2
replaceStep <-	Replace workflow step	SYSargsList
results	SYSargs accessor methods	SYSargs
returnRPKM	Convert read counts to RPKM values	data.frame
rmdPath	LineWise accessor methods	LineWise
run_DESeq2	Run DESeq2	count df and contrasts

Table S3: List of functions and methods defined by *systemPipeR* (not complete). As new functions continue to be developed, this list is expected to grow over time.

Functions	Description	Class of input
run_edgeR	Run edgeR	count df and contrasts
runCommandline	Execute SYSargs and SYSargs2	SYSargs/SYSargs2
runDiff	Runs differential abundance analysis	SYSargs2
runInfo	Execution status of each step	SYSargsList
runWF	Execute workflow	SYSargsList
updateWF	Update SYSargs2 object	SYSargs2
SampleName	Extract sample IDs from workflows	SYSargs2 and SYSargsList
scaleRanges	Scale spliced ranges to genome coordinates	GRangesList
seeFastq	Quality reports for FASTQ files	paths to FASTQ files
seeFastqPlot	Plot seeFastq result	list
setlist	List of ID vectors	list
software	SYSargs accessor methods	SYSargs
SPRproject	Initialize workflow project	paths to directories
status	SYSargs2/LineWise accessor methods	SYSargs2/LineWise
statusWF	SYSargsList accessor methods	SYSargsList
stepName	SYSargsList/LineWIse accessor methods	SYSargsList/LineWise
stepsWF	SYSargsList accessor methods	SYSargsList
subset	Subset workflow by samples	SYSargsList
subsetParam	Accessories function to modify the Command-line	SYSargs2
subsetWF	Subsetting SYSargs2 class slots	SYSargs2
symLink2bam	Symbolic links for IGV	SYSargs/SYSargs2
sysargs	SYSargs accessor methods	SYSargs
sysargs2	SYSargs2 accessor methods	SYSargs2
sysargslist	SYSargsList accessor methods	SYSargsList
SYSargsList	Constructs SYSargsList object	SYSargsList
systemArgs	SYSargs object from param and targets files	SYSargs
targets	SYSargs2 accessor methods	SYSargs2
targets.as.df	Convert targets to list or data.frame	list or data.frame
targetsheader	SYSargs/SYSargs2 accessor methods	SYSargs/SYSargs2
targetsin	SYSargs accessor methods	SYSargs
targetsout	SYSargs accessor methods	SYSargs
targetsWF	SYSargsList accessor methods	SYSargsList
tryCL	Test whether command-line software is installed	name of software
tryPath	Test if path exists	character vector
variantReport	Generate variant report	paths to VCF files
varSummary	Summary statistics of variants	paths
vennlist	Return venn intersects	VENNset
vennPlot	Plot Venn diagram	VENNset
viewEnvir	Return names of objects in SYSargsList environment	SYSargsList
wf	SYSargs2 accessor methods	SYSargs2
writeParamFiles	Write cwl/yml files generated from command-line string	SYSargs2
writeTargetsout	Write updated targets out to file	SYSargs or SYSargs2
writeTargetsRef	Write targets file with reference information	path
yamlinput	SYSargs2/SYSargsList accessor methods	SYSargs2/SYSargsList
yamlinput<-	Replacement methods	SYSargs2/SYSargsList
createWF	Create a SYSargs2	SYSargs2
write.clt	Write clt to file	SYSargs2
write.yml	Write yml to file	SYSargs2
cmdTool2wf	Write cwl to file	SYSargs2

References

[1] YAML The Official YAML Web Site. `https://yaml.org/` (2021) Accessed: 2024-2-25.

[2] Xie, Y., Allaire, J. J., and Grolemund, G. (2018) R markdown: The definitive guide, Chapman and Hall/CRC, .

[3] Lang, M., Bischl, B., and Surmann, D. (feb, 2017) batchtools: Tools for R to work on batch systems. *The Journal of Open Source Software,* (10).

[4] Bischl, B., Lang, M., Mersmann, O., Rahnenführer, J., and Weihs, C. (2015) BatchJobs and BatchExperiments: Abstraction Mechanisms for Using R in Batch Environments. *Journal of Statistical Software,* **64**(11), 1–25.
